# Is overall and timing-specific physical activity associated with depression in older adults?

**DOI:** 10.3389/fpubh.2023.1241170

**Published:** 2023-09-22

**Authors:** Jiaren Chen, Ting-Fu Lai, Li-Jung Lin, Jong-Hwan Park, Yung Liao

**Affiliations:** ^1^Graduate Institute of Sport, Leisure and Hospitality Management, National Taiwan Normal University, Taipei, Taiwan; ^2^Department of Health Promotion and Health Education, National Taiwan Normal University, Taipei, Taiwan; ^3^Health Convergence Medicine Laboratory, Biomedical Research Institute, Pusan National University Hospital, Busan, Republic of Korea

**Keywords:** different proportions of physical activity, accelerometer, circadian rhythm, depression, mental health

## Abstract

**Objective:**

Regarding the circadian rhythm regulating an individual’s response to external stimulation, it remains unclear whether older adults engaged in physical activity at different times of day may be differently related to depression symptoms. Thus, this study aimed to investigate the association between overall and timing-specific physical activity and depressive symptoms in older Taiwanese adults.

**Methods:**

This cross-sectional study was conducted at a medical center in Taipei City, Taiwan, between September 2020 and December 2021. The participants were community-dwelling older adults aged ≥65 who could walk independently and were not at high risk of cognitive dysfunction. Physical activity was measured using a triaxial accelerometer (GT3X+, ActiGraph) and categorized into timing-specific periods (morning: 06:01–12:00; afternoon: 12:01–18:00; evening: 18:01–24:00) as well as overall physical activity, which included both light physical activity (LPA) and moderate-to-vigorous physical activity (MVPA). A 15-item Geriatric Depression Scale was utilized to assess and measure depressive symptoms. Multivariate linear regression models were estimated for data analysis.

**Results:**

A total of 180 older adults (55.0% female; 80.5 ± 7.1 years old; 18.3% at risk of depression) were included. On average, the participants spent 237.3 (± 85.7) minutes in LPA per day and 12.8 (± 17.0) minutes in MVPA per day. The results showed that overall MVPA engagement was associated with lower depressive-symptom scores [*B* = −1.357, 95% CI (−2.561, −0.153)] in older adults. However, no significant associations were observed between overall LPA, timing-specific MVPA and LPA, and depression in older adults.

**Conclusion:**

To prevent depression in older adults, it is advisable to accumulate a higher amount of MVPA throughout the entire day rather than engage in LPA. Further studies employing a prospective design are necessary to validate and strengthen our findings.

## 1. Introduction

Several nations worldwide are entering an aging society as a consequence of demographic changes. By 2050, it is anticipated that one in six individuals globally will be aged 65 years or older. Additionally, it is projected that Taiwan will achieve the status of a super-aged society by 2025 (1). Consequently, there is an increasing number of health issues among the older population, including depression, which is listed by the World Health Organization as among the world’s three most prevalent diseases, along with heart disease and cancer (2). Additionally, depression is more prevalent in old age and could lead not only to higher mortality rates but also to an economic and medical burden on society (3). Therefore, there is an urgent need to develop effective strategies to prevent depression in older adults.

In recent years, a growing number of studies have confirmed the positive benefits of physical activity (PA) in the prevention of depression among older adults (4). PA has been recognized as the most fundamental chronobiological tool and a potential zeitgeber for the human circadian-rhythm system (5). Circadian rhythms play a crucial role in health, exerting significant effects on aging, longevity, and disease pathogenesis, and are dynamic over an individual’s lifespan (6, 7). These rhythms become increasingly disrupted as aging progresses, due to factors such as changes in the endocrine system and sleep patterns, causing a variety of negative health conditions (8). Conversely, different intensities and timing of PA stimuli can regulate a circadian rhythm and help maintain chronobiological homeostasis, regulate metabolism, prevent certain diseases, and ultimately improve physical health, including physical and mental aspects (9). Indeed, engaging in PA at different times of the day may alter metabolism and the circadian rhythm, thus changing different disease conditions in some cohorts (10). In recent studies, the health benefits of PA have been found to differ depending on whether the activity is performed in the morning, afternoon, or evening (11, 12). In addition, it is worth noting the inconsistent results regarding which time periods and PA intensities are optimal for preventing depression in older adults. For example, PA in the morning is beneficial to weight loss (13) and improves metabolism (14), while exercise in the afternoon or evening improves blood glucose (15). Nevertheless, regardless of timing, overall moderate-to-vigorous-intensity physical activity (MVPA) is related to favorable physical function (16) and lower risks of all-cause, cardiovascular-disease, and cancer mortality (17). In summary, most of these previous studies on the association between PA at different times and health focused on physical aspects (13–18); only one study was based on a population of older adults (16), while limited studies have objectively used PA measurements (16, 17). Using objective measurement (i.e., triaxial accelerometers), total and timing-specific PA and its intensity PA can be accurately captured throughout the day to avoid recall and social-desirability biases, especially in the older adult population (19–21).

Given that light and fresh air are possible mediators of the circadian rhythm, there may be synergistic effects. We hypothesized that engagement in MVPA during the morning and afternoon was negatively associated with older adults’ depressive-symptom scores. Therefore, this study focused on investigating the association between total or timing-specific PA and depression among community-dwelling older adults in Taiwan.

## 2. Methods

### 2.1. Participants and study design

This cross-sectional study collected data from community-dwelling older adults aged ≥65 years who could walk independently and were not at risk of cognitive dysfunction. Data were collected using convenience sampling from September 2020 to December 2021. Participants with chronic diseases such as cardiovascular disease and neurological issues were not excluded as the recruitment site was the Department of Geriatrics and Gerontology (DGG) at National Taiwan University (NTU) Hospital (NTUH). A doctor from the NTUH’s DGG outpatient department assessed whether the participants met the criteria and explained the study process. The sample participants provided written informed consent and were informed of the detailed implementation measures. The participants completed self-reported questionnaires on sociodemographic status, health status, and lifestyle behaviors. PA data were collected using a tri-accelerometer worn on the hip for 7 consecutive days. Valid data were defined as those collected from participants who wore the acceleration protocol for at least 10 h during waking hours on at least four valid days. Depression scores were calculated using the Chinese version of the 15-item Geriatric Depression Scale (GDS-15) (22). At the end of the study, the participants received a US$7 gift voucher as a token of our appreciation. This research received ethical approval from the Research Ethics Committee of the NTUH (REC number: 202008046RINC). We recruited a total of 300 participants for the first phase of this study. The exclusion criteria were as follows: (1) participants with a high risk of cognitive dysfunction after screening by the Mini-Mental State Examination (MMSE) (*n* = 51); (2) participants who failed to complete the self-reported questionnaire (*n* = 36); and (3) the participants’ accelerometer data did not meet the valid-wear criterion, which was at least 4 valid days (including three weekdays and one weekend day) and at least 10 h/day (*n* = 33). After the data were screened, 180 participants were included in the final sample for the study.

### 2.2. Measures

#### 2.2.1. Geriatric depressive symptoms

The GDS-15 was used to assess depressive symptoms in the older adults. This scale has been shown to have good validity and reliability, with a Cronbach’s *α* value of 0.81 (23); its validity and reliability were also demonstrated based on a sample of Taiwanese older adults (22). The GDS-15 comprises 15 questions and is easy for older adults to complete. Total scores range from 0 (lower depressive symptoms) to 15 (higher depressive symptoms) points. A total score of ≥5 points indicates the presence of clinical depressive symptoms, while a score of <5 points indicates no depressive symptoms (24).

#### 2.2.2. PA

A waist-worn triaxial accelerometer (ActiGraph GT3X+, Pensacola, FL, United States) was used to measure the times spent in LPA (100–2019 counts/min) and MVPA (≥ 2020 counts/min) (25). The accelerometer was not worn during water-related activities, such as swimming, and the participants were requested to record that time in their diaries. Following previous studies (26), we identified the different time intervals for PA: (a) morning: 06:01–12:00; (b) afternoon: 12:01–18:00; and (c) evening: 18:01–24:00. The accelerometer data showed that approximately 90% of the participants slept between 0:00 and 06:00 for each night in this study. The ActiLife software version 6.0 was used to calculate the accelerometer data; all the data were processed using 60 s epochs of sampling frequency at the default setting (i.e., 30 Hz).

#### 2.2.3. Covariates

We collected data on sociodemographic characteristics, health status, and lifestyle behaviors using self-rated questionnaires. Sociodemographic characteristics included age, sex (female and male), marital status, employment, living status, and education level (either university-education level or none). Health status included body-mass index (BMI), chronic diseases (< 4 and ≥4), diabetes, hypertension, hyperlipidemia, and depressive symptoms (no and yes). BMI was calculated as weight in kilograms divided by height in meters squared and was divided into four groups based on the official standard of the Taiwan Ministry of Health and Welfare: underweight (< 18.5 kg/m^2^), normal (18.5–23.9 kg/m^2^), overweight (24–26.9 kg/m^2^), and obese (≥ 27 kg/m^2^). Depressive symptoms were assessed using the GDS-15, with a cut-off point of <5 points indicating no depressive symptoms and that ≥5 points indicating the presence of depressive symptoms. Lifestyle behaviors included whether they had a habit of smoking and alcohol use. Monitor wear time was assessed by the accelerometer.

### 2.3. Statistical analyses

Descriptive statistical analysis was conducted to obtain the means and standard deviations (SDs) of the sample characteristics (*n* = 180), such as socio-demographic factors (age, sex, educational level, and BMI), lifestyle behaviors (smoking, alcohol use, high blood pressure, high blood cholesterol, and diabetes), GDS-15 scores, LPA, and MVPA (Table 1). Multiple linear regression models were used to estimate the association between the time spent in timing-specific (in percentage) and overall LPA/MVPA (in minutes) based on the GDS-15 using 95% confidence intervals (CI) and unstandardized coefficients (B); *p* < 0.05 was considered significant. Binary logistic regression analyses were also used to estimate the association between overall and timing-specific PA with depression risk. Age, sex, education level, BMI, living alone, smoking, drinking, high blood pressure, diabetes, high blood cholesterol, and total accelerometer wear time were controlled for as confounders. All statistical analyses in this study were conducted using IBM SPSS Statistics 23.0, while *p* < 0.05 indicated the significance level.

**Table 1:** Characteristic of the participants (*n* = 180).

Categorical variables	*n*	%
*Age*		
65–74	46	25.6
≥75	134	74.4
*Sex*		
Female	99	55
Male	81	45
*Education level*		
No graduate level	101	56.1
Graduate and higher level	79	43.9
*Living conditions*		
Alone	15	8.3
Another	165	91.7
*Chronic diseases*		
Fewer than four	154	85.6
Four and above	26	14.4
*Smoking*		
No	164	91.1
Yes	16	8.9
*Alcohol use*		
No	158	87.8
Yes	22	12.2
*Depressive risk*		
No	147	81.7
Yes	33	18.3
*BMI (kg/m^2^)*		
Underweight	4	2.2
Normal	92	51.1
Overweight	46	25.6
Obesity	38	21.1

Continuous variables	Mean	SD
*Light physical activity*		
Overall (min/day)	237.3	85.7
During the morning^a^^,^^d^ (%)	35.1	10.5
During the afternoon^b^^,^^d^ (%)	37.8	6.7
During the evening^c^^,^^d^ (%)	25.0	8.8
*Moderate-to-vigorous physical activity*		
Overall (min/day)	12.8	17
During the morning^a^^,^^d^ (%)	37.8	28.1
During the afternoon^b^^,^^d^ (%)	36.1	25.2
During the evening^c^^,^^d^ (%)	24.6	28.1
Wear time (min/day)	867.6	79.2
Total sleep time (min/day)	449.8	69.6

BMI: body mass index; SD: standard deviation.

a During the morning: 06:01–12:00.

b During the afternoon: 12:01–18:00.

c During the evening: 18:01–24:00.

d The percentages of LPA and MVPA during the morning, afternoon, and evening do not add up to 100.0% because each percentage was based on an average of 180 participants.

## 3. Results

Table 1 presents the characteristics of the 180 older adults (55.0% female; 80.5 ± 7.1 years old). Of the participants, 74.4% were over 75 years of age, 55% were women, 43.9% had university or higher education level, 91.7% participants lived with others, 91.1% were nonsmokers, 87.8% did not consume alcohol, 51.1% had a normal BMI, 85.6% had fewer than four chronic diseases, and 18.3% were at risk of depression. On average, the participants spent 237.3 (± 85.7) minutes in LPA per day and 12.8 (± 17.0) minutes in MVPA per day.

Table 2 presents the associations between timing-specific and overall LPA/MVPA and depressive-symptom scores. In the adjusted model, the overall MVPA duration was significantly associated with lower total depressive symptom scores [*B* = −1.357, 95% CI (−2.561, −0.153), *p* < 0.05]. However, timing-specific LPA [during the morning: *B* = −2.321, 95% CI (−7.034, 2.391), *p* > 0.05; during the afternoon: *B* = 4.617, 95% CI (–1.760, 10.994), *p* > 0.05; during the evening: *B* = −1.711, 95% CI (−6.635, 3.214), *p* > 0.05], timing-specific MVPA [during the morning: *B* = −1.080, 95% CI (−2.677, 0.518), *p* > 0.05; during the afternoon: *B* = 1.593, 95% CI (−0.069, 3.255), *p* > 0.05; during the evening: *B* = −0.285, 95% CI (−1.902, 1.332), *p* > 0.05], and even overall LPA [*B* = −0.009, 95% CI (−0.394, 0.376), *p* > 0.05] were not significantly associated with the total depressive symptom scores. Binary logistic regression analysis results also showed no significant associations between overall and timing-specific PA with depression risk in older individuals (Supplementary Table S1).

**Table 2:** Associations of overall and timing-specific light physical activity/moderate-to-vigorous physical activity with depressive scores (*n* = 180).

Outcome	Overall LPA			Percentage of overall LPA								
				Morning			Afternoon			Evening		
	*B*	95% CI	*p*	*B*	95% CI	*p*	*B*	95% CI	*p*	*B*	95% CI	*p*
	−0.009	(−0.394, 0.376)	0.963	−2.321	(−7.034, 2.391)	0.332	4.617	(−1.760, 10.994)	0.155	−1.711	(−6.635, 3.214)	0.494

Total score of depressive symptoms	Overall MVPA			Percentage of overall MVPA								
				Morning			Afternoon			Evening		
	*B*	95% CI	*p*	*B*	95% CI	*p*	*B*	95% CI	*p*	*B*	95% CI	*p*
	−1.357	(−2.561, −0.153)	0.027*	−1.080	(−2.677, 0.518)	0.184	1.593	(−0.069, 3.255)	0.060	−0.285	(−1.902, 1.332)	0.728

*B* unstandardized linear regression coefficient; CI, confidence interval. All the models were adjusted for age, sex, BMI, living alone, smoking, drinking, high blood pressure, diabetes, high blood cholesterol, and total accelerometer wear time. ^*^p < 0.05.

## 4. Discussion

To our knowledge, this is the first study to utilize an accelerometer to objectively examine the relationship between overall and timing-specific PA and depression in Taiwanese older adults. The most important finding of this study is that accumulation in MVPA, but not in LPA, throughout the day, regardless of different timing, was significantly associated with lower depressive symptom scores in older adults. This finding is significant for intervention designers and public health practitioners in designing effective programs for older adults to prevent depression.

This study provided two pieces of evidence. The first evidence is that accumulation in total PA throughout the day rather than the specific timing of PA is more optimal for depression prevention, which contradicts our hypothesis based on the circadian rhythm. Several previous studies have found that engaging in PA at different times of the day may be related to different physical-health outcomes (13–15, 18, 27). Our findings are similar to those obtained in two studies (16, 17) that indicated that accumulating more overall MVPA duration was more important than timing for physical function (16) and mortality risks (17).

A possible reason for the unexpected results is that this study did not investigate the participants’ activity patterns (outdoor or indoor), illumination time, and dietary behavior. Previous studies have reported that depression may be related to other behavior (e.g., a diet that reduces inflammation in the brain by consuming anti-inflammatory substances, indirectly lowering the risk of mental illness (28)) and environmental factors (e.g., light exposure promotes the synthesis of vitamin D, which acts as a hormone to stimulate a positive emotion) (29). Consequently, this may explain why various time periods for PA are not significant, and the fundamental mechanism could be explored in future work.

Another important finding is that engaging in MVPA rather than LPA was more important for depression prevention in older adults. It is well-established that engaging in MVPA is associated with lower risks of depression among older adults (30, 31) because MVPA could contribute to psychological (distraction and self-efficacy), social (social support and social interaction), and physiological (increase in endorphins) benefits (32). A possible reason for LPA not being related to depression is that compared with MVPA, LPA is more likely to come from standing, housekeeping, or slow walking, which is likely to be less stimulating to the brain (33). Therefore, it is possible that older adults who engage in at least a moderate intensity of PA may produce sufficient stimulation to the brain, which then activates the production of endorphins and increases serum endocrine concentrations, with a tendency toward homogeneous pleasure responses (34, 35). Consequently, these findings imply that MVPA may play a crucial protective role against depressive symptoms in older adults.

A strength of the present study is that the measurement of PA was objective and under free-living environments. Additionally, our study is the first attempt to examine the associations of different timings for PA with mental health. Our data aid public policy research on overall PA and depression, which requires gathering and applying solid evidence on PA and depression in the future. However, the results should be interpreted in the context of several limitations. First, the sample of participants recruited clinically was not representative of the entire community-dwelling population, as it included a smaller number of participants, roughly half of whom were female, and 18% displayed risks of depression. Future research will necessitate a more extensive and representative sample to investigate the relationship between time-specific and overall MVPA and depression in older adults. Second, the study excluded the time periods between 0:00 a.m. and 6:00 a.m., which were tacitly recognized as sleep time. This might have reduced the time included in the calculation of physical activities. However, our data indicate that approximately 90% of the participants have overlapping sleep patterns covering the sleep-time period. Third, due to the cross-sectional design of the study, a causal relationship between the percentage and duration of time-specific and total MVPA and depression cannot be inferred. Finally, The GDS-15, used in this study, evaluates depressive symptoms over the past week, limiting its capacity to reflect longer-term psychological changes. To better understand the evolving nature of depression in older adults, future studies should consider methods like ecological momentary assessment.

## 5. Conclusion

This study demonstrated that engaging in total MVPA was associated with the prevention of depression in adults, regardless of the time of day that PA was performed. It is proposed that future depression-prevention initiatives for older adults focus on a sufficient intensity of PA accumulation throughout the day.

## Data availability statement

The original contributions presented in the study are included in the article/Supplementary material, further inquiries can be directed to the corresponding authors.

## Ethics statement

The study commenced following the participants’ signing of an informed-consent contract. The procedure in this study was approved by the Research Ethics Committee of the NTUH (REC number: 202008046RINC). All the methods included in this study are in accordance with the declaration of Helsinki.

## Author contributions

JC: conceptualization, writing original draft, and formal analysis. T-FL: conceptualization, writing-review and editing, and formal analysis. L-JL: writing-review, editing, and supervision. J-HP: writing-review, editing, and funding acquisition. YL: conceptualization, writing-review, editing, funding acquisition, and supervision. All authors contributed to the article and approved the submitted version.

## Abbreviations

BMI: Body-mass index
CI: Confidence interval
DGG: Department of Geriatrics and Gerontology
GDS-15: 15-item Geriatric Depression Scale
LPA: light physical activity
MVPA: moderate-to-vigorous physical activity
NTU: National Taiwan University
NTUH: National Taiwan University Hospital
PA: Physical activity
