# Supplementary material for: Is overall and timing-specific physical activity associated with depression in older adults?
Source: Front Public Health. 2023 Sep 22;11:1241170. doi: 10.3389/fpubh.2023.1241170 (PMC10560127; doi:10.3389/fpubh.2023.1241170)
Supplement: Supplementary file 1 [file Table_1.docx]

**Supplementary Table S1 Associations of overall and timing-specific LPA/MVPA with depression risk (n = 180)**

| **Outcome** | **Overall LPA** | | | **Percentage of overall LPA** | | | | | | | | |
| --- | --- | --- | --- | --- | --- | --- | --- | --- | --- | --- | --- | --- |
|  |  |  |  | **Morning** | | | **Afternoon** | | | **Evening** | | |
|  | **OR** | **95%CI** | ***p*** | **OR** | **95%CI** | ***p*** | **OR** | **95%CI** | ***p*** | **OR** | **95%CI** | ***p*** |
| **Depression risk** | 1.022 | (0.721, 1.448) | 0.902 | 0.666 | (0.016, 28.501) | 0.832 | 319.638 | (0.607, 168443.92) | 0.071 | 0.100 | (0.001, 11.475) | 0.341 |
| **Outcome** | **Overall MVPA** | | | **Percentage of overall MVPA** | | | | | | | | |
|  |  |  |  | **Morning** | | | **Afternoon** | | | **Evening** | | |
|  | **OR** | **95%CI** | ***p*** | **OR** | **95%CI** | ***p*** | **OR** | **95%CI** | ***p*** | **OR** | **95%CI** | ***p*** |
| **Depression risk** | 0.122 | (0.013, 1.141) | 0.065 | 1.052 | (0.227, 4.878) | 0.948 | 4.616 | (0.839, 25.393) | 0.079 | 0.386 | (0.067, 2.237) | 0.289 |

OR: odds ratio; CI: confidence interval.
All the models were adjusted for age, sex, education level, drinking, high blood pressure, diabetes, high blood cholesterol, and accelerometer wear time.

**p* < 0.05.
